# Incentives and Disincentives for the Treatment of Depression and Anxiety: A Scoping Review

**DOI:** 10.1177/070674371405900706

**Published:** 2014-07

**Authors:** Rachelle Ashcroft, Jose Silveira, Brian Rush, Kwame McKenzie

**Affiliations:** 1 Postdoctoral Fellow, Social Aetiology of Mental Illness Training Program, Centre for Addiction and Mental Health, Toronto, Ontario; Assistant Professor, School of Social Work, Renison University College, University of Waterloo, Waterloo, Ontario.; 2 Chief of Psychiatry, Medical Director, Mental Health and Addiction Program, St Joseph’s Health Centre, Toronto, Ontario; Assistant Professor, Department of Psychiatry, University of Toronto, Toronto, Ontario.; 3 Senior Scientist, Health Equity Research Group, Social and Epidemiological Research Department, Centre for Addiction and Mental Health, Toronto, Ontario; Professor, Department of Psychiatry, University of Toronto, Toronto, Ontario; Associate Professor, Dalla Lana School of Public Health, University of Toronto, Toronto, Ontario.; 4 Medical Director of Underserved Populations Program, Centre for Addictions and Mental Health, Toronto, Ontario; Professor of Psychiatry, University of Toronto: Director of Division of Equity, Gender and Populations, Toronto, Ontario; Director of Canadian Institutes of Health Research Social Aetiology of Mental Illness Training Program, Toronto, Ontario; President, Canadian Mental Health Association Toronto, Toronto, Ontario.

**Keywords:** anxiety, depression, primary care, treatment, incentives, disincentives

## Abstract

**Objective:**

There is widespread support for primary care to help address growing mental health care demands. Incentives and disincentives are widely used in the design of health care systems to help steer toward desired goals. The absence of a conceptual model to help understand the range of factors that influence the provision of primary mental health care inspired a scoping review of the literature. Understanding the incentives that promote and the disincentives that deter treatment for depression and anxiety in the primary care context will help to achieve goals of greater access to mental health care.

**Method:**

A review of the literature was conducted to answer the question, how are incentives and disincentives conceptualized in studies investigating the treatment of common mental disorders in primary care? A comprehensive search of MEDLINE, PsycINFO, CINAHL, and Google Scholar was undertaken using Arksey and O’Malley’s 5-stage methodological framework for scoping reviews.

**Results:**

We identified 27 studies. A range of incentives and disincentives influence the success of primary mental health care initiatives to treat depression and anxiety. Six types of incentives and disincentives can encourage or discourage treatment of depression and anxiety in primary care: attitudes and beliefs, training and core competencies, leadership, organizational, financial, and systemic.

**Conclusions:**

Understanding that there are 6 different types of incentives that influence treatment for anxiety and depression in primary care may help service planners who are trying to promote improved mental health care.

## Abstract (fr)

**Objectif:**

L’idée que les soins de première ligne aident à répondre aux demandes croissantes de soins de santé mentale obtient un large soutien. Les incitatifs et les désincitatifs sont largement utilisées dans la conception des systèmes de santé pour aider à prendre la direction des buts escomptés. L’absence d’un modèle conceptuel destiné à comprendre la variété de facteurs qui influencent la prestation de soins de santé mentale de première ligne a inspiré une revue étendue de la littérature. Comprendre les incitatifs qui favorisent le traitement de la dépression et de l’anxiété dans le contexte des soins de première ligne ainsi que les désincitatifs qui les découragent contribuera à atteindre les objectifs d’accès accru aux soins de santé mentale.

**Méthode:**

Une revue de la littérature a été menée pour répondre à la question : comment les incitatifs et les désincitatifs sont-ils conceptualisées dans les études portant sur le traitement des troubles mentaux communs dans les soins de première ligne? Une recherche détaillée de MEDLINE, PsycINFO, CINAHL et Google Scholar a été entreprise à l’aide du cadre méthodologique en 5 stades d’Arksey et O’Malley pour les revues étendue.

**Résultats:**

Nous avons repéré 27 études. Une variété d’incitatifs et de désincitatifs influencent la réussite des initiatives de soins de santé mentale de première ligne pour traiter la dépression et l’anxiété. Six types d’incitatif et de désincitatifs peuvent encourager ou décourager le traitement de la dépression et de l’anxiété dans les soins de première ligne : attitudes et croyances, formation et compétences essentielles, leadership, organisationnelles, financières, et systémiques.

**Conclusions:**

Savoir qu’il existe 6 différents types d’incitatifs qui influencent le traitement de l’anxiété et de la dépression dans les soins de première ligne peut aider les planificateurs de services qui tentent de promouvoir de meilleurs soins de santé mentale.

Depression and anxiety contribute significantly to the global burden of disease.^1^,^2^ Also known as common mental disorders (CMDs), they are a leading mental health cause of disability^2^ and a major cause of morbidity and mortality.^3^,^4^ There is agreement that the best way to respond to the population need for treatment of CMDs is to develop capacity in primary care, but this often does not happen.^3^,^5^–^8^ Understanding factors that may promote or deter the treatment of CMDs will be helpful to achieve goals of greater access to mental health care.^9^–^11^

There are various, different incentives and disincentives that influence health care systems.^11^ In health care, an incentive refers to a motivator that influences the action of professionals, teams, and organizations.^9^–^11^ A disincentive can be something that acts as an intentional or unintentional deterrent that discourages action.^12^,^13^ Identification and elimination of disincentives that create barriers to a particular service provision may be necessary to achieve various goals of a health care system.^14^,^15^

There are a broad range of incentives that may be active in primary care. Incentives may motivate individual physicians, including: professional expectations, ethics, norms, regulations, altruism, autonomy, intellectual satisfaction, desire to promote health and well-being of patients, and financial incentives.^16^–^18^ Organizational incentives may include the following: culture, size, health care provider composition, and financial levers.^19^

Although incentives are omnipresent in health care,^24^ little is known about them, or disincentives, when it comes to the provision of mental health care in primary care. We were unable to identify a conceptual model that helps to explain the range of incentives and disincentives that influence primary mental health care. Understanding these factors may help providers to develop mental health care capacity in primary care. This scoping review does not advocate for any one method of treatment; instead, it aims to advance our knowledge and to aid providers who wish to facilitate change and develop treatment for CMDs in primary care.

## Methods

Scoping reviews aim to map key concepts, sources, and types of evidence that underpin a research area.^21^ A scoping review is necessary when literature on a topic is being assembled for the first time, and (or) when the topic under investigation is complex or nonhomogeneous.^22^ Although there is a growing body of relevant literature, evidence related to incentives and disincentives in primary mental health care has not been systematically compiled.

**Clinical Implications**

- A combination of the 6 types of incentives may be helpful for clinicians wanting to improve or make changes to mental health care for depression and anxiety in primary care.
- Mitigating strategies may be required to counter the effects of disincentives.

**Limitations**

- Our scoping review included and summarized only 27 studies.
- Studies included in the scoping review were restricted to English-language publications.
- Unlike systematic reviews, the quality of studies is not assessed.

The 5-stage methodological framework of Arksey and O’Malley^21^ guides this scoping review:

1. identify research question;
2. identify relevant results;
3. study selection;
4. charting data; and
5. report results.

The question guiding the scoping review is, how are incentives and disincentives conceptualized in studies investigating the treatment of CMDs in primary care?

At stage 2, an a priori search strategy was developed in consultation with the senior librarian at the Centre for Addiction and Mental Health to identify the breadth of the peer-reviewed literature related to incentives and disincentives and treatment of CMDs in primary care. A search from inception through April 2013, restricted to the English language, was conducted within MEDLINE, PsycINFO, CINAHL, and Google Scholar to identify all articles with content inclusive of CMDs, primary care, incentives and (or) disincentives. Key words were searched within 3 groups using “OR,” then groups 1 to 3 were combined using “AND.” Searches included a combination of 3 groups of terms:

1. primary care, primary health care, family physician*, general practi*;
2. common mental disorder*, mental disorder*, anxiety, depressi*; and
3. incentiv*, disincentiv*, buy-in, motivat*, organization* change, knowledge translation, knowledge integrat*; integrat*, implement*, and implementation research.

Duplicates of articles were removed.

Stage 3 examined abstracts of all articles selected to identify those that met all 4 of the inclusion criteria:

1. article was a research study;
2. primary care was referred to in the abstract (inclusive of all primary care terminology);
3. the words CMDs, anxiety, depression, or general mental health disorders were found in the abstract; and
4. the word incentive(s) or disincentive(s) was used in the abstract, or mention was made of practice or organizational change. The reason for this is because incentives and disincentives are associated with practice or organizational change.

A full text review was completed of all articles selected for the final sample. A chart was developed that guided identification of key areas relevant for the scoping review. What follows are results from the scoping review, examining incentives, disincentives, and the treatment of CMDs in primary care.

## Results

Following these steps generated a breadth of results. MEDLINE, PsycINFO, and CINAHL generated 1377 results, and Google Scholar generated more than 2000 results. Results generated from Google Scholar were extensively reviewed until a decision was made that remaining results seemed unrelated to topic area. Titles and abstracts of these articles were reviewed to identify articles related to the topic. From the above, 96 articles were selected. We also conducted footnote-chasing and reference-checking in the selected articles, which generated an additional 10 articles for inclusion. The sample of 106 articles then went through a second round of scrutiny to determine inclusion and exclusion to the final sample.

**Figure 1:**
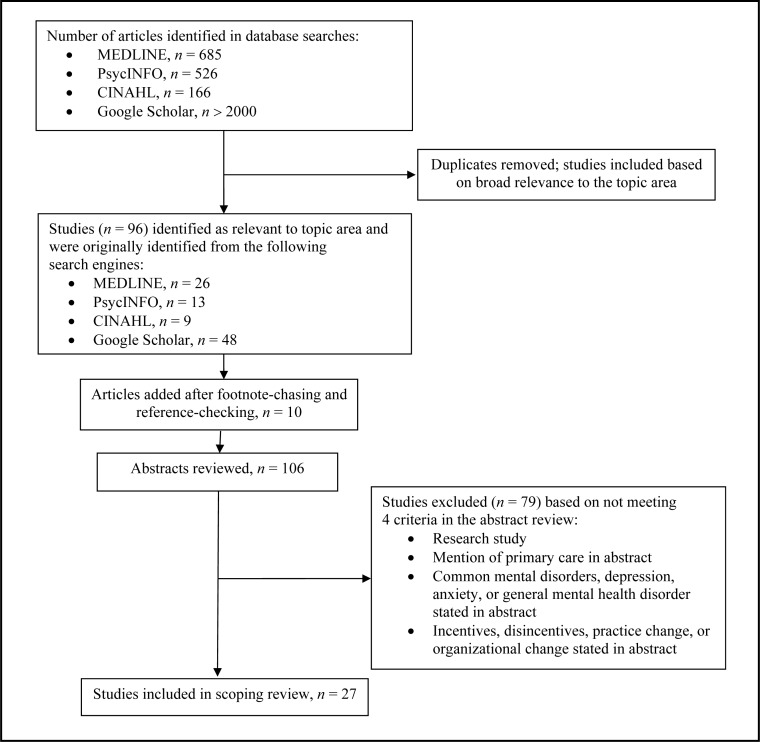
Methodology used to conduct scoping review

Among the 106 articles identified for review, 27 met all 4 inclusion criteria (Figure 1).^23^–^49^ Articles were excluded after abstract review because 49 were not research studies; 2 did not refer to primary care; 6 did not refer to CMDs; and 21 did not refer to incentives, disincentives, provider change, or organizational change. Although 1 study did meet all 3 criteria, it was later excluded because it presented results of a study examining incentives and disincentives of primary care providers’ participation in a practice audit rather than the provision of mental health care.

### Study Publication Year, and Region

There was a range of quantitative methodologies (14 studies), mixed methodologies (6 studies), qualitative methodologies (5 studies), and reviews of the literature (2 studies). Research studies spanned from 1985 to 2012, with 55% published between the years of 2008 and 2012. This suggests that incentives and disincentives in primary mental health care is a fairly recent topic of examination.

**Figure 2:**
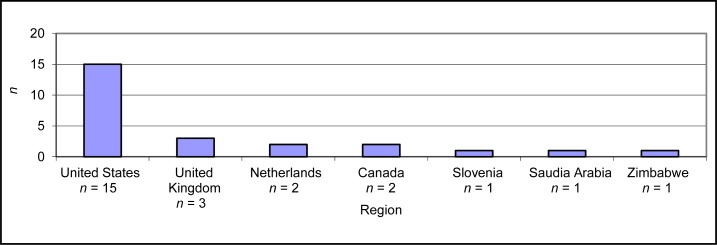
Geographical regions represented in scoping review sample

There was a range of geographical regions represented in the studies. Two studies^33^,^34^ performed literature reviews, thus potentially drawing on data that spanned various geographical locations, and thus are not included in Figure 2.

### Treatment of Common Mental Disorders in Primary Care

All articles included in the review had a central focus on depression, anxiety, CMDs, or general mental health disorders (online eTable 1: Twenty-seven articles included in scoping review^23^–^49^). Depression was a focus in 70% (19 articles) of the studies, anxiety in 14% of the studies (4 articles), and general mental disorders in 18% of the studies (5 articles). Some studies had more than 1 mental health focus, resulting in the number of mental health disorders being greater than the total of 27 individual studies. Only 2 studies included both depression and anxiety.^38^,^46^

### Incentives and Disincentives

Two criteria were used to identify incentives: the term incentive was used, and (or); there was mention of something that encouraged or helped to facilitate individual practitioners and (or) organizations to implement mental health care practices of any type. Two criteria were used to identify disincentives: the term disincentive was used, and (or); there was mention of something that deterred implementation of mental health care practices of any type. Consistent with a scoping review, these data were charted to identify themes.^21^ Themes for incentives were derived separately from themes for disincentives. Going into the scoping review, we did not anticipate that the themes for incentives would be the same as the themes for disincentives.

All 27 studies referred to incentives and (or) disincentives, although the terminology of incentives and disincentives may not have been used. For example, 55% (15 studies)^23^,^24^,^28^,^29^,^34^,^35^,^37^,^40^–^42^,^44^–^47^,^49^ used the term incentive at least once, and only 11% (3 studies)^24^,^40^,^48^ used the term disincentive at least once.

A range of incentives were identified, including financial (55% of studies) and nonfinancial (41% of studies). Incentives aimed at people appeared in 74% of the sample (20 studies), and organizational incentives appeared in 45% of the sample (11 studies). Disincentives were identified in 59% (16 studies) of the sample. Incentives and disincentives identified in the sample span 6 themes: attitudes and beliefs, training and core competencies, leadership, organizational, financial, and systemic (Table 2).

## Six Types of Incentives and Disincentives

### Attitudes and Beliefs

Attitudes and beliefs can encourage people^26^,^29^,^36^ and organizations^24^,^29^ to provide primary mental health care. Attitudes and beliefs refer to a personal motivation or intention to provide care for people with mental disorders.^26^,^29^ For example, Curran et al^29^ state that following through on a recommended anxiety management strategy was

most plentiful when physicians . . . and nurses had enthusiastically ‘bought in’ to the intervention. The factor most linked to strong buy-in was a belief that mental health concerns should be a priority.^p 6^

Kirchner et al^36^ agree that attitudes and beliefs of primary care practitioners affect success of new mental health initiatives. Attitudes and beliefs that shape organizational culture facilitates successful implementation of new treatment programs; for example, the Coordinated Anxiety Learning and Management (CALM) intervention described by Curran et al.^29^

Attitudes and beliefs of primary care providers can act as a disincentive to the treatment of CMDs. Curran et al^29^ suggest that a “buy-in barrier”^p 6^—or the absence of mental health as a care priority—may deter certain practices, such as referring to specialists. A lack of physician interest in treating anxiety disorders was also identified as a deterrent to the successful implementation of a new anxiety treatment program.^29^

### Training and Core Competencies

Adequate training and development of personal knowledge and skills also helps encourage treatment for CMDs.^26^,^27^,^39^,^43^ Bilsker et al^27^ demonstrated that physician training led to substantial implementation of depression interventions. Roškar et al^43^ showed that physician training on the recognition and treatment of depression influenced prescription practices. Nease et al^39^ demonstrated that a 9-month training program on key elements of depression care and practice change strategies led to a significant increase in the implementation of treatment and the management of depression in various primary care organizations.

Inadequate training or skills may also deter primary mental health care.^23^ Abas et al^23^ suggest that substandard training poses a challenge to depression treatment:

The standard training in mental health for PHC [primary health care] staff . . . lasted only 16 hours and did not include a psychiatrist—who may have been able to provide additional information and a better perspective on the use of antidepressants.^p 164^

Abas et al^23^ suggest that inadequate training hinders what they consider to be optimal screening and pharmaceutical management.

### Leadership

Leadership was the third theme of incentives identified in the sample. Nease et al^39^ demonstrated that the significant increase in depression treatment was, in part, due to champion leadership. Kirchner et al^36^ stated that leadership, supportive of a new mental health program, helps others to adapt to the change process and fosters success in program implementation. Meredith et al^37^ also found that leadership was a key factor that influenced successful implementation and maintenance of quality improvement efforts for the treatment of depression in primary care.

The absence of leadership may deter efforts to implement new programs.^36^ Curran et al^29^ stated that “many physicians reported that enthusiasm for the intervention could wane without supportive attention from ‘champions’ or ‘opinion leaders.’”^p 5^ Bauer et al^25^ demonstrated that inadequate clinical leadership from psychiatrists for consultation or supervision was a deterrent to effective treatment and management of depression in primary care.

### Organizational

Organizational incentives are the fourth type of incentive that help to encourage primary mental health care.^23^,^36^,^37^,^39^ Organizational structures^37^ can help encourage treatment for CMDs by including career advancement options for mental health work.^23^

Organizational factors can also act as a deterrent. One study that used the term disincentive specifically sought to identify disincentives by interviewing primary care physicians on factors impeding care for patients diagnosed with anxiety.^48^ Time constraints were cited as a disincentive to physicians’ clinical practices by limiting their ability to carry out care in a way that was consistent with recommended guidelines.^48^ Another study, by Coventry et al,^28^ considered guidelines that emphasize single diseases to be a deterrent to comprehensive mental health care.

In their evaluation of a collaborative care project of depression treatment at community health centres, Bauer et al^25^ identified several organizational constraints that may deter the treatment of CMDs in primary care. Having a preexisting way of providing care for mental disorders acted as a deterrent in the implementation of a new depression treatment program.^25^ Bauer et al^25^ found that insufficient staffing deterred the provision of follow-up. The exclusion of options for career advancement for mental health work was cited as an additional organizational disincentive.^23^ Lastly, Fleury et al^30^ found that the lack of incentives to promote cooperation with mental health care professionals ended up being a disincentive to the implementation of collaborative mental health care.

### Financial

Financial incentives are the fifth type of incentives identified in the sample.^30^ Financial incentives can influence priorities for care.^28^,^30^ For example, financial incentives were described as a means to motivate physicians to provide care for people with mental disorders and to encourage adoption of particular treatment modalities.^30^ Fleury et al^30^ recommend financial incentives to be aimed at primary care physicians to increase overall management of mental disorders. As well, financial incentives can encourage organizations to implement particular health care practices.^34^ For example, Katon and Seelig^34^ suggest providing “incentives for health care organizations to implement enhanced screening for depression and evidence-based collaborative care programs.”^p 465^ Qureshi et al^42^ also stated that “capitation provides true incentives for integrating mental health into primary care.”^p 904^ Nevertheless, Unützer et al^46^ suggest payfor-performance incentives for the treatment of CMDs.

Coventry et al^28^ consider financial incentives that assume a more generic approach instead of emphasizing a single disease as more effective in promoting treatment for mental health disorders such as depression. Conversely, Steel et al^44^ described a strong association between financial incentives for specific ailments and quality of clinical care. Toner et al^45^ suggest that financial incentives may be effective in encouraging adherence to depression guidelines by focusing incentives on treatment outcomes and screening.

Financial incentives were considered a disincentive for several reasons. Financial incentives can deter focus away from a particular aspect of treatment for mental disorders. For example, Hoebert et al^32^ demonstrated that a reimbursement restriction led to a decrease in diagnosis and initiation of benzodiazepines for patients diagnosed with anxiety. Post et al^41^ demonstrated that performance incentives included various health conditions but did not extend to depression care. Further, incurring additional financial costs can also be a disincentive. Kessler et al^35^ stated that out-of-pocket expenditures and insurance policies that have strict coverage limitations can also be a deterrent.

### Systemic

Systemic incentives were also identified in the sample as being influential. Williams et al^49^ describe how access to mental health resources influences practice patterns and has the “potential to increase identification of . . . mental health disorders in primary care.”^p 429^ Increasing access to mental health resources may help to subdue any reluctance that primary care providers have to identify mental health conditions.^49^

Systemic disincentives can also act to deter treatment.^37^,^47^,^49^ Williams et al^49^ suggest that the lack of access to mental health resources results in the reluctance of primary care providers to identify mental health conditions. To a broader extent, poor coordination between the primary care and the mental health care systems was also considered a deterrent for mental health care.^47^

## Conclusion

There are 6 types of incentives and disincentives that may encourage or discourage the treatment of CMDs in primary care: attitudes and beliefs, training and core competencies, leadership, organizational, financial, and systemic. Although financial incentives are important to the integration of mental health in primary care,^50^–^52^ this review shows that they can also act as a disincentive.

Incentives and disincentives are used in the design of health care systems and assist to leverage change.^10^,^11^,^15^,^16^,^53^,^54^ Results indicate that health professionals are motivated by more than financial incentives.^11^,^19^ Designing health care systems that encourage treatment of CMDs in primary care may require some combination of the 6 different types of incentives. To promote treatment of CMDs in primary care we need to consider how the 6 types of disincentives may be working individually or in combination to prevent that from happening. Simply educating primary care physicians about the identification and treatment of CMDs has not been successful to change practices.^55^ The influence of incentives and disincentives may help to explain why primary care physicians experience such difficulties when attempting to implement practice changes to better manage CMDs.^56^

Changes to organizational structures of care can help improve outcomes for depression.^56^ Understanding the way that disincentives work may provide some explanation to barriers preventing these changes from occurring.^56^ A gap uncovered in this review is the lack of attention on disincentives. Only 3 articles^24^,^40^,^48^ even mention the word disincentives. Identification and elimination of disincentives that act as barriers to care is essential to provide quality care in primary care.^14^,^15^ This is an area that needs greater attention to follow through with strengthening of treatment for CMDs in primary care. Canada is a leader in primary care transformation, yet this review suggests that it is lagging behind when it comes to generating knowledge on a key tool used to guide change in health care systems.

This scoping review has helped to conceptualize incentives and disincentives influential to primary mental health care, but further investigation is required to provide an understanding of how it is that the range of incentives and disincentives interact and influence the provision of mental health care. To help develop capacity for treating CMDs in primary care and respond to the overwhelming burden of disease, research examining the range of incentives and disincentives is encouraged.

## 



## Figures and Tables

**Table 2:** Incentives and disincentives identified in scoping review^23^–^49^

Variable	As incentives	As disincentives
Attitudes and beliefs	Bao et al^24^; Benzer et al^26^; Curran et al^29^; Kirchner et al^36^	Curran et al^29^; Kirchner et al^36^
Training and core competencies	Abas et al^23^; Benzer et al^26^; Bilsker et al^27^; Nease et al^39^; Roškar et al^43^; Williams et al^49^	Abas et al^23^
Leadership	Bao et al^24^; Holm and Severinsson^33^; Kirchner et al^36^; Meredith et al^37^; Nease et al^39^; Nutting et al^40^	Bauer et al^25^; Curran et al^29^; Kirchner et al^36^; Nutting et al^40^
Organizational	Abas et al^23^; Curran et al^29^; Kirchner et al^36^; Nease et al^39^; Meredith et al^37^	Abas et al^23^; Bauer et al^25^; Coventry et al^28^; Fleury et al^30^; Holm and Severinsson^33^; Kirchner et al^36^; Nutting et al^40^; van Boeijen et al^48^
Financial	Bao et al^24^; Coventry et al^28^; Fleury et al^30^; Grembowski et al^31^; Hoebert et al^32^; Katon and Seelig^34^; Kessler et al^35^; Meyer et al^38^; Post et al^41^; Qureshi et al^42^; Steel et al^44^; Toner et al^45^; Unützer et al^46^; Upshur^47^; Williams et al^49^	Bao et al^24^; Coventry et al^28^; Hoebert et al^32^; Kessler et al^35^; Nutting et al^40^; Post et al^41^
Systemic	Williams et al^49^	Meredith et al^37^; Upshur^47^; Williams et al^49^
