# Supplementary material for: Incentives and Disincentives for the Treatment of Depression and Anxiety: A Scoping Review
Source: Can J Psychiatry. 2014 Jul;59(7):385–92. doi: 10.1177/070674371405900706 (PMC4086319; doi:10.1177/070674371405900706)
Supplement: Supplementary file 1 [file CJP-2014-vol59-July-385-392-eTable1.pdf]

Ashcroft R, Silveira J, Rush B, et al. Incentives and disincentives for the treatment of depression and anxiety: a scoping review. Can J Psychiatry. 2014;59(7):385–392.

Table 1: 27 studies included in scoping review [23-49]

Author	Year	Region	Purpose	Key Findings
Abas e al.	2003	Zimbabwe	To examine if training depression training program influenced practice changes; to help inform future training and policy development	Training not as effective as anticipated
Bao et al.	2011	US	To examine if Collaborative Care Depression activities reflected the evidence-based protocol	Substantial variation in caseload across health organizations and in CCD resource use
Bauer et al.	2011	US	To evaluate collaborative care project for depression treatment	Organizations differed dramatically in quality indicators and outcome measures
Benzer et al.	2012	US	To develop theory to conceptualize barriers and facilitators to mandated coordinated mental health care	Leadership and organizational factors can be barriers and facilitators to primary mental health care
Bilsker et al.	2012	Canada	To determine physician uptake of two brief interventions for depression; and, to determine patients’ adherence to self-management	Substantial implementation of depression intervention after training
Coventry et al.	2011	UK	To identify barriers for managing depression in primary care in people with diabetes and chronic health disorders	Case-finding approaches, practitioners' limited knowledge, and normalization of depression are barriers to recognition and treatment of depression
Curran et al.	2012	US	To identify facilitators/barriers to implementing and sustaining an anxiety management program	Barriers and facilitators are: provider attitudes/behaviours, clinic structure, intervention characteristics, patient characteristics
Fleury et al.	2012	Canada	To assess collaboration between GPs and mental healthcare providers, identify factors that enable and hinder integrated care	Barriers to management of pts with mental disorders include: lack of resources, long wait times, lack of training, time constraints, no incentives for collaboration; and inappropriate physician payment models
Grembowski et al.	2005	US	To determine association between managed care, physician job satisfaction, and quality of care; To determine if physician job satisfaction linked with health outcomes	Physician job satisfaction linked to some but not all measures of pt-rated quality of primary care; physician job satisfaction not linked to health outcomes

Hoebert et al.	2012	Netherlands	To assess impact of reimbursement restriction on benzodiazepine use in patients with newly diagnosed anxiety or sleeping disorder in PC	Proportion of patients prescribed a benzodiazepine lower after policy restriction implemented in 2009 than in 2008 for both anxiety and sleeping disorder
Holm and Severinsson	2012	-	To identify barriers and facilitators of success when implementing Chronic Care Model for management of depression in primary care	Various organizational barriers exist
Katon, and Seelig	2008	-	To conduct analysis of collaborative care studies in literature	Collaborative care improves: antidepressant adherence, depressive outcomes, patient satisfaction, and primary care satisfaction
Kessler et al.	1985	US	To test use of General Health Questionnaire (GHQ) as a mental illness/emotional distress screening tool	Pt characteristics influence prediction of diagnostic and management behaviour, clinician and setting characteristics may influence patient-provider interaction
Kirchner et al.	2004	US	To gain a better understanding of critical components for implementing integrated mental health care services in rural VA community-based outpatient clinics	Leadership, staff attitudes and beliefs, organizational factors, and community factors influence success of implementing mental health care initiatives
Meredith et al.	2006	US	To assess factors for successful implementation and maintenance of quality improvement efforts for treatment of depression in primary care	All sites made changes toward improving care in three of six categories: delivery system redesign, self-management strategies, and information systems
Meyer	2000	US	To investigate role of physician characteristics, practice organization, and managed care financial structures in practice variation for evaluation of common problems in primary care	Financial incentives not associated with an effect on costs of diagnostic evaluation
Nease et al.	2008	US	To test feasibility and effectiveness of a modified improvement collaborative approach to improving depression care	Significant change in primary care practices after champion leaders participated in depression training program
Nutting et al.	2007	US	To characterize experiences of those attempting to implement and sustain the depression model at multiple levels of five Health Care Organizations	Successful implementation related to a broadly shared vision and commitment among all levels of the organization

Post et al.	2009	US	To describe a framework of PC organizational factors and depression management	Variation exists in organizational factors influencing implementation of evidence-based depression management
Qureshi et al.	2001	Saudi Arabia	To examine how a two-week psychiatric training course for primary care physicians helps increase likelihood of integrating treatment of mental disorders	Training increases support for treating mental disorders in primary care, value multidisciplinary approaches, providers require more knowledge for treatment and management of mental disorders
Roškar et al.	2010	Slovenia	To implement and evaluate an educational program for primary care physicians on recognition and treatment of depression and suicide rates	Training group had increase in prescription rates for antidepressants; Training assisted physicians to recognize and manage depression; no change in suicide rates
Steel et al.	2007	UK	To compare incentivised and non-incentivised indicators of quality of care	Significant increase occurred for indicators linked to incentive payments
Toner et al.	2010	UK	To assess primary care physicians adherence to NICE guidelines for managing depression in adults	Physicians follow NICE guidelines when screening pts with physical illness and less likely when there is no presence of a physical illness
Unützer et al.	2011	US	To examine changes in quality of care and patient outcomes observed among MHIP participants before and after implementation of pay for performance incentive program	Pay for performance incentive improved quality and outcomes such as: improvement in depression severity, and time to improvement was reduced
Upshur	2005	US	To determine practice level issues and concerns of implementing mental health care in PC	Need for depression treatment high in primary care; accessing behavioral health care services challenging
van Boeijen	2005	Netherlands	To compare effectiveness and feasibility of guided self-help, the Anxiety Disorder Guidelines of the Netherlands College of General Practitioners, and CBT	All three treatments showed improvement. Feasibility of the Anxiety Disorder Guidelines was low compared with self-help; Self-help most desirable for physicians because of time constraints
Williams et al.	2007	US	To assess changes in pediatricians' behavioral health practices after structured interventions	Changes in all 80 clinics include: use of new measurement tool, trained and hired care managers, and development of ongoing relationship with a psychiatrist
